# Supplementary material for: Comparison of four low-cost carbapenemase detection tests and a proposal of an algorithm for early detection of carbapenemase-producing Enterobacteriaceae in resource-limited settings
Source: PLoS One. 2021 Jan 12;16(1):e0245290. doi: 10.1371/journal.pone.0245290 (PMC7802922; doi:10.1371/journal.pone.0245290)
Supplement: Attachment — Submitted filename: Response to Reviewers.docx [file pone.0245290.s001.docx]

Dr. Y. S. Wijayasinghe

Department of Biochemistry and Clinical Chemistry

Faculty of Medicine / University of Kelaniya

PO Box 06, Thalagolla Road

Ragama

Sri Lanka

[supunw@kln.ac.lk](mailto:supunw@kln.ac.lk)

December 03, 2020

The Editor

PLOS ONE Journal

Dear Professor Iddya Karunasagar,

**Rebuttal letter (PONE-D-20-31489)**

We thank you and the reviewers for thorough reading and constructive feedback of our manuscript entitled “Comparison of four low-cost carbapenemase detection tests and a proposal of an algorithm for early detection of carbapenemase-producing *Enterobacteriaceae* (CPE) in resource-limited settings.” for the publication in your prestigious journal. We were glad to have the opportunity to revise and resubmit the manuscript.

The manuscript has been revised after careful consideration of the editor and reviewer comments. After making suggested changes, we believe the quality of the manuscript is now improved. Hence, we are pleased to submit the revised research article for further consideration in PLOS ONE journal. On the following pages, you will find our response to each point raised by the reviewers. On behalf of all authors, I thank you for consideration of this resubmission. We appreciate your time and look forward to your response.

Sincerely yours,

Y.S. Wijayasinghe, PhD (Toledo, USA)

--

Corresponding author

PONE-D-20-31489
Comparison of four low-cost carbapenemase detection tests and a proposal of an algorithm for early detection of carbapenemase-producing Enterobacteriaceae in resource-limited settings.
PLOS ONE

Dear Dr. Wijayasinghe,

Thank you for submitting your manuscript to PLOS ONE. After careful consideration, we feel that it has merit but does not fully meet PLOS ONE’s publication criteria as it currently stands. Therefore, we invite you to submit a revised version of the manuscript that addresses the points raised during the review process.

The manuscript requires substantial revision in all sections

Please submit your revised manuscript by Jan 04 2021 11:59PM. If you will need more time than this to complete your revisions, please reply to this message or contact the journal office at plosone@plos.org. When you're ready to submit your revision, log on to https://www.editorialmanager.com/pone/ and select the 'Submissions Needing Revision' folder to locate your manuscript file.

Please include the following items when submitting your revised manuscript:

- A rebuttal letter that responds to each point raised by the academic editor and reviewer(s). You should upload this letter as a separate file labeled 'Response to Reviewers'.
- A marked-up copy of your manuscript that highlights changes made to the original version. You should upload this as a separate file labeled 'Revised Manuscript with Track Changes'.
- An unmarked version of your revised paper without tracked changes. You should upload this as a separate file labeled 'Manuscript'.

If you would like to make changes to your financial disclosure, please include your updated statement in your cover letter. Guidelines for resubmitting your figure files are available below the reviewer comments at the end of this letter.

If applicable, we recommend that you deposit your laboratory protocols in protocols.io to enhance the reproducibility of your results. Protocols.io assigns your protocol its own identifier (DOI) so that it can be cited independently in the future. For instructions see: <http://journals.plos.org/plosone/s/submission-guidelines#loc-laboratory-protocols>

We look forward to receiving your revised manuscript.

Kind regards,

Iddya Karunasagar
Academic Editor
PLOS ONE

Journal Requirements:

When submitting your revision, we need you to address these additional requirements.

1. Please ensure that your manuscript meets PLOS ONE's style requirements, including those for file naming. The PLOS ONE style templates can be found at

https://journals.plos.org/plosone/s/file?id=wjVg/PLOSOne_formatting_sample_main_body.pdf and

https://journals.plos.org/plosone/s/file?id=ba62/PLOSOne_formatting_sample_title_authors_affiliations.pdf

 We have reformatted the manuscript according to the given PLOSOne guidelines.

2. Please provide more information on how another researcher could obtain the CRE strains used in this study so that they could reproduce your results.

Thank you for your suggestion. “All data are fully available without restriction” is already mentioned in the Data Availability section in the manuscript submission form.

If further explanation is needed, following statement can be added.

Materials of the current study are available from the corresponding author upon a reasonable request.

3. To comply with PLOS ONE submission guidelines, in your Methods section, please provide additional information regarding your statistical analyses. In addition, please report your p-values to support your claims. For more information on PLOS ONE's expectations for statistical reporting, please see https://journals.plos.org/plosone/s/submission-guidelines.#loc-statistical-reporting.

Thank you for your comment. We have added additional information on statistical analysis in the Materials and Methods.

Additional Editor Comments (if provided):

Two reviewers have commented on the manuscript and made several pertinent points to improve the manuscript. Please address these point by point. The number of NDM resistant strains used is small and conclusions based on such small number should have a caveat.

We appreciate the reviewer’s constructive feedback on our manuscript.

We agree with the editor that our study contains a smaller number of NDM producers. We have added several sentences in the discussion section to highlight the limitations of our study.

“Although, this study consisted of a small sample of NDM producing CRE (n=4), these findings are in agreement with previous studies in which MHT has been found to have poor sensitivity (50 - 86%) for detection of MBLs (18, 28) and low specificity (91%) (18).” (Line no 244-246 in manuscript with track changes)

“Since the present study is mainly based on the OXA-48-like carbapenemase producing CRE, further studies with a large sample of CP-CRE isolates with adequate representation of commonly found genotypes (eg: KPC, NDM, OXA-48-like, IMP, VIM, etc.) may help better understanding of the performance of the proposed algorithm.” (Line no 330-334 in manuscript with track changes)

[Note: HTML markup is below. Please do not edit.]

Reviewers' comments:

Reviewer's Responses to Questions

**Comments to the Author**

1. Is the manuscript technically sound, and do the data support the conclusions?

The manuscript must describe a technically sound piece of scientific research with data that supports the conclusions. Experiments must have been conducted rigorously, with appropriate controls, replication, and sample sizes. The conclusions must be drawn appropriately based on the data presented.

Reviewer #1: Partly

Reviewer #2: Yes

2. Has the statistical analysis been performed appropriately and rigorously?

Reviewer #1: No

Reviewer #2: Yes

3. Have the authors made all data underlying the findings in their manuscript fully available?

The [PLOS Data policy](http://www.plosone.org/static/policies.action#sharing) requires authors to make all data underlying the findings described in their manuscript fully available without restriction, with rare exception (please refer to the Data Availability Statement in the manuscript PDF file). The data should be provided as part of the manuscript or its supporting information, or deposited to a public repository. For example, in addition to summary statistics, the data points behind means, medians and variance measures should be available. If there are restrictions on publicly sharing data—e.g. participant privacy or use of data from a third party—those must be specified.

Reviewer #1: Yes

Reviewer #2: Yes

4. Is the manuscript presented in an intelligible fashion and written in standard English?

PLOS ONE does not copyedit accepted manuscripts, so the language in submitted articles must be clear, correct, and unambiguous. Any typographical or grammatical errors should be corrected at revision, so please note any specific errors here.

Reviewer #1: Yes

Reviewer #2: Yes

5. Review Comments to the Author

Please use the space provided to explain your answers to the questions above. You may also include additional comments for the author, including concerns about dual publication, research ethics, or publication ethics. (Please upload your review as an attachment if it exceeds 20,000 characters)

**Reviewer #1:** The manuscript entitled “Comparison of four low-cost carbapenemase detection tests and a proposal of an algorithm for early detection of carbapenemase-producing Enterobacteriaceae in resource-limited settings” describes the four different phenotypic methods (MHT, mCIM, CNPt, and Carba NP-direct) for carbapenemase detection and also proposed a protocol to simplify rapid detection of CP-CRE using the combination of CNPt-direct and mCIM. While the study brings information, required improvements, including statistical interpretation.

*Major comments*
Statistical interpretation of the results is not clearly mentioned in the manuscript, especially to compare among four low-cost carbapenemase detection tests that were used in this study.

Thank you for your comment. We have added additional information on statistical analysis in the Materials and Methods and have included 95% confidence intervals and *P* values in the results section.

Lines no 101-103: Details of the control microorganisms can be mentioned.

Thank you for the suggestion. We have added the details of the control organisms and have rewritten the section on bacterial isolates in the materials and methods.

Line 163-164: Statistics analysis used for the study can be explained in detail, including the software used, if any.

Thank you for your suggestion. We have added the relevant information.

Line 188 and table 2: The microorganisms used in this study, Enterobacteriaceae species, has not been isolated in this study. Hence, it can be mentioned with reference(s).
The proposed protocol for rapid detection of CP-CRE (Line 302) using the combination of CNPt-direct and mCIM, can be move to the result section, followed by discussion of the same in the discussion section.

Thank you for your comment. We have revised the Bacterial isolates in the methods section. Following sentence is mentioned with reference 20.

A total of 117 previously characterized *Enterobacteriaceae* clinical isolates were tested to evaluate the performance of four phenotypic carbapenemase detection assays, namely MHT, mCIM, CNPt, and CNPt-direct (20).

Reference no 20:

Kumudunie WGM, Wijesooriya LI, Namalie KD, Sunil-Chandra NP, Wijayasinghe YS. Epidemiology of multidrug-resistant Enterobacteriaceae in Sri Lanka: First evidence of blaKPC harboring Klebsiella pneumoniae. Journal of infection and public health. 2020;13(9):1330-5.

We think that there is some overlap between results and discussion sections. Therefore, we prefer to keep the proposed algorithm at the present location as it is the take home message of the manuscript.

*Minor comments*
Lines no: 51-52: Please rephrase the sentences

Thank you for your suggestion. We have rephrased the sentence.

Line no 59: microorganisms, instead “organisms.”

Replaced

Lines no 68-72: These two sentences are contrary to each other. The first/second sentence can be rephrased differently.

Thank you for pointing out. We have rephrased the second sentence as follow.

However due to high cost, unavailability of necessary instrumentation and technical expertise in many diagnostic laboratories, the PCR is not suitable for routine detection of CP-CRE in developing countries.

Line no 88: Expand the abbreviation, MHT, as appears for the first time

Thank you for pointing out. “modified Hodge test” is first appeared in Line 80 (Line 77 in original submission), so MHT was added next to it.

Line 93: Which “molecular methods” are authors talking about?

Thank you for pointing out. By “molecular methods” we broadly referred the techniques used to detect the presence of particular genes. To be more specific, we have replaced the “molecular method” with “PCR based detection methods”.

Lines 111, 122, 126, 137, 140, 153, 154: remove “ - ” between number and unit

Removed

Lines 210-222: This paragraph can be summarised.

Thank you for your suggestion. We have reduced the paragraph.

Lines 221-222: This sentence can be removed from here

Agreed. The sentence was removed.

Lines 224-226: Include a reference

Agreed. Two references (Ref 26 and 27) were added.

Line 226: ….we observed overall low sensitivity…….

This sentence was revised as,

we observed relatively low overall sensitivity (90.7%) and specificity (92.1%), and low positive predictive value (56.0%) for MHT (Table 1).

Lines 227-228: Rephrase this sentence

Thank you for your suggestion. We have rephrased the sentence.

**Reviewer #2:** Dear Authors
Th paper compares 4 techniques for detecting carbapenem resistance in CRE organisms. The paper compares the sensitivity, specificity, positive predictive value and negative predictive value for 4 methodologies as a comparison.
The paper brings out important problems in various methods for detecting carbapenamases. But the following can be added

1. What were the clinical sources of the isolates? The choice of antibiotic is also dependent on the site of infection hence this data could enhance the data in the paper

Thank you for your comment. The information (sources, species and genetic identification, antibiotic susceptibilities, etc.) regarding the clinical isolates used in this study has been previously published and is available in reference 20 (an open access article). Reference 20 is cited in the materials and methods.

Reference no 20:

Kumudunie WGM, Wijesooriya LI, Namalie KD, Sunil-Chandra NP, Wijayasinghe YS. Epidemiology of multidrug-resistant Enterobacteriaceae in Sri Lanka: First evidence of blaKPC harboring Klebsiella pneumoniae. Journal of infection and public health. 2020;13(9):1330-5.

2. This is a pilot study kind of data as far as the numbers are concerned. For example, 4 NDM isolates were only present in the study. It would be important to explain that larger number of isolates may help in understanding the shortcomings of these tests better

We agree that our study contained only a very low number of NDM isolates. Even with this low numbers, similar trend was observed when compared to the published studies. Therefore, the following sentences were added to the discussion section in the manuscript.

“Although, this study consisted of a small sample of NDM producing CRE (n=4), these findings are in agreement with previous studies in which MHT has been found to have poor sensitivity (50 - 86%) for detection of MBLs (18, 28) and low specificity (91%) (18).” (Line no 244-246 in manuscript with track changes)

“Since the present study is mainly based on the OXA-48-like carbapenemase producing CRE, further studies with a large sample of CP-CRE isolates with adequate representation of commonly found genotypes (eg: KPC, NDM, OXA-48-like, IMP, VIM, etc.) may help better understanding of the performance of the proposed algorithm.” (Line no 330-334 in manuscript with track changes)

3. When the authors mention Oxa-48 producers or NDM producers, how were these characterized? It would be important to also have this mentioned in the paper

Thank you for the suggestion. We have revised the Bacterial isolates in the Materials and Methods with more details.

A total of 117 previously characterized *Enterobacteriaceae* clinical isolates were tested to evaluate the performance of four phenotypic carbapenemase detection assays, namely MHT, mCIM, CNPt, and CNPt-direct (20). These strains included 54 genetically identified CP-CRE producing carbapenemases of Ambler class A. B, and D: KPC (n=2), NDM (n=4), OXA-48-like (n=44), NDM+OXA-48-like (n=4). Three non-CP-CRE and 60 non-CRE consisted of 30 extended-spectrum β-lactamase producing *Enterobacteriaceae* (ESBL-PE: ESBL production was identified by double disc synergy test using 30 μg cefotaxime and 20/10 μg amoxicillin-clavulanic acid discs according to CLSI guidelines) and 30 non-ESBL-PE ( *Enterobacteriaceae* sensitive to both third generation cephalosporins and carbapenems) served as negative control in the study.

The detailed genetic characterization of the CP-CRE used in this study is available at

Reference no 20:

Kumudunie WGM, Wijesooriya LI, Namalie KD, Sunil-Chandra NP, Wijayasinghe YS. Epidemiology of multidrug-resistant Enterobacteriaceae in Sri Lanka: First evidence of blaKPC harboring Klebsiella pneumoniae. Journal of infection and public health. 2020;13(9):1330-5.

4. Why were other CRO's like Pseudomonas or Acinetobacter not studied?

Thank you for your comment. We understand that it is worthwhile to explore the carbapenem resistance in *Pseudomonas* and *Acinetobacter* as these CRO’s are also public health concerns. However, this manuscript is a product of a government funded, ongoing study on carbapenem resistant *Enterobacteriaceae*. The tests we studied in this research cannot be directly employed to detect other CRO’s. Therefore, the study of the carbapenem resistance in *Pseudomonas* and *Acinetobacter* would be the next step of our research.

6. PLOS authors have the option to publish the peer review history of their article ([what does this mean?](https://journals.plos.org/plosone/s/editorial-and-peer-review-process#loc-peer-review-history)). If published, this will include your full peer review and any attached files.


If you choose “no”, your identity will remain anonymous but your review may still be made public.


**Do you want your identity to be public for this peer review?** For information about this choice, including consent withdrawal, please see our [Privacy Policy](https://www.plos.org/privacy-policy).

Reviewer #1: No

Reviewer #2: **Yes:** Dr. Anusha Rohit

[NOTE: If reviewer comments were submitted as an attachment file, they will be attached to this email and accessible via the submission site. Please log into your account, locate the manuscript record, and check for the action link "View Attachments". If this link does not appear, there are no attachment files.]

While revising your submission, please upload your figure files to the Preflight Analysis and Conversion Engine (PACE) digital diagnostic tool, <https://pacev2.apexcovantage.com/>. PACE helps ensure that figures meet PLOS requirements. To use PACE, you must first register as a user. Registration is free. Then, login and navigate to the UPLOAD tab, where you will find detailed instructions on how to use the tool. If you encounter any issues or have any questions when using PACE, please email PLOS at [figures@plos.org](mailto:figures@plos.org). Please note that Supporting Information files do not need this step.
